# Airway administration of corticosteroids for prevention of bronchopulmonary dysplasia in premature infants: a meta-analysis with trial sequential analysis

**DOI:** 10.1186/s12890-017-0550-z

**Published:** 2017-12-15

**Authors:** Zhi-Qun Zhang, Ying Zhong, Xian-Mei Huang, Li-Zhong Du

**Affiliations:** 1 Department of Neonatology, the Children’s Hospital, Zhejiang University School of Medicine, No. 3333 Bingsheng Road, Hangzhou City, Zhejiang 310002 China; 2 Department of Pediatrics, Hangzhou First People’s Hospital, Nanjing Medical University, No. 261 Huansha Road, Hangzhou City, Zhejiang 310002 China

**Keywords:** Inhaled corticosteroids, Bronchopulmonary dysplasia, Preterm infants, Meta-analysis, Neurodevelopmental outcomes

## Abstract

**Background:**

Uncertainly prevails with regard to the use of inhalation or instillation steroids to prevent bronchopulmonary dysplasia in preterm infants. The meta-analysis with sequential analysis was designed to evaluate the efficacy and safety of airway administration (inhalation or instillation) of corticosteroids for preventing bronchopulmonary dysplasia (BPD) in premature infants.

**Methods:**

We searched MEDLINE, EMBASE, CINAHL, and Cochrane CENTRAL from their inceptions to February 2017. All published randomized controlled trials (RCTs) evaluating the effect of airway administration of corticosteroids (AACs) vs placebo or systemic corticosteroid in prematurity were included. All meta-analyses were performed using Review Manager 5.3.

**Results:**

Twenty five RCTs retrieved (*n* = 3249) were eligible for further analysis. Meta-analysis and trial sequential analysis corrected the 95% confidence intervals estimated a lower risk of the primary outcome of BPD (relative risk 0.71, adjusted 95% confidence interval 0.57–0.87) and death or BPD (relative risk 0.81, adjusted 95% confidence interval 0.71–0.97) in AACs group than placebo and it is equivalent for preventing BPD than systemic corticosteroids. Moreover, AACs fail to increasing risk of death compared with placebo (relative risk 0.90, adjusted 95% confidence interval 0.40–2.03) or systemic corticosteroids (relative risk 0.81, 95% confidence interval 0.62–1.06).

**Conclusions:**

Our findings suggests that AACs (especially instillation of budesonide using surfactant as a vehicle) are an effective and safe option for preventing BPD in preterm infants. Furthermore, the appropriate dose and duration, inhalation or instillation with surfactant as a vehicle and the long-term safety of airway administration of corticosteroids needs to be assessed in large trials.

**Electronic supplementary material:**

The online version of this article (10.1186/s12890-017-0550-z) contains supplementary material, which is available to authorized users.

## Background

Bronchopulmonary dysplasia (BPD), defined as oxygen dependence at 36 weeks of postmenstrual age (PMA), is a severe complications of extremely premature infants. The reported morbidity of BPD ranges from 24% in preterm infants born at 28 weeks to 79% in preterm infants born at 23 weeks [1].The survival infants of BPD are at high risk for long-term injury to both lung and brain [2–4]. Pulmonary inflammation plays a central, modulating role in the pathogenesis of BPD [5–7]. It has been confirmed that corticosteroids have strong anti-inflammatory effects. Randomized controlled trials (RCTs) have shown that systemic administration of corticosteroids reduces the incidence of BPD but aggravate short-term and long-term adverse effects [8–11]. Theoretically, airway administration (inhalation or instillation) of corticosteroids, on the other hand, demonstrate high pulmonary deposition, low systemic bioavailability, and rapid systemic clearance, thereby reducing the adverse effects [12, 13].

A range of reviews address the use of inhaled corticosteroids for preventing BPD [14, 15]. Onland et al. [14] have attempted the late use (≥7 days)) of inhaled corticosteroids to reduce BPD incidence in preterm infants and concluded that such treatment do not reduce the outcomes of BPD and death or BPD. Similarly, Shah et al. [15] also concluded that early (<2 weeks) administration of inhaled corticosteroids was ineffective for reducing the incidence of BPD in ventilated very low birth weight premature infants. However, some overlap in ages at administration of the inhaled corticosteroid existed in the included studies in their meta-analysis. In recent, a large RCT confirmed that inhalation of budesonide reduces both BPD and persistent ductus arteriosus (PDA) [16]. However, there was a trend toward increased mortality. Subsequently, a meta-analysis including the RCT by Bassler D et al. [17] showed that inhaled corticosteroids reduced risk for BPD and had no effect on death. Their meta-analysis included several therapeutic studies and failed to evaluate the intratracheal instillation of steroids [18, 19]. In addition, when valuating for the outcome of death, the meta-analysis included a study published as an abstract, which had a large impact on the results [17, 20]. The authors did not carry out sensitive analysis to determine the stability of the results [17]. In addition, Cochrane reviews also have compared inhaled and systemic corticosteroids [19, 21, 22]. Most of the main outcome were concluded from a small number of the studies with few infants providing data [21, 22].

Uncertainty prevails with regard to the use of inhalation or instillation steroids to prevent BPD in preterm infants [23]. Recently, 2 RCTS about airway administration of corticosteroids (AACs) for preventing BPD and neurodevelopmental outcomes have been published [24, 25]. Therefore, to better understand the potential efficacy and safety of AACs for reducing the risk of BPD or/and death in preterm infants, as compared with placebo or systemic corticosteroids, we performed an up-to-date systematic review of published RCTs. We verified the robustness and reliability of our findings by implementing trial sequential analysis and sensitivity analyses.

## Methods

### Study identification

This systematic review was conducted and is reported according to the recommendations of the Preferred Reporting Items for Systematic Reviews and Meta-Analyses (PRISMA) statement [26]. Electronic searches were carried out in multiple databases, including PubMed, Web of Science, Embase, Cochrane Library, Clinicaltrials.gov, Controlled-trials.com, Google scholar, VIP, WangFang and proceedings of the Pediatric Academic Society meetings (from 1980) on December 31, 2016 for relevant studies. Search terms included: preterm infant, premature infant, infant-newborn, bronchopulmonary dysplasia, chronic lung diseases, anti-inflammatory agents, neurodevelopmental outcomes, steroids, glucocorticoids, corticosteroids; administration, inhalation; aerosols, budesonide, beclomethasone, dipropionate, flunisolide, and fluticasone propionate. No language restriction was applied. The protocol of this systematic review was registered before the literature search in PROSPERO (Prospero2016 CRD42016054098) [27].

### Eligibility criteria

Studies had to meet the following criteria: 1) randomized controlled trials; 2) infants were randomized to receive treatment with an airway administration (inhalation or instillation) of corticosteroid vs placebo or systemic corticosteroids; 3) reported more than one of the following outcome parameters: primary outcome of BPD (defined by the need for supplemental oxygen or positive pressure support at 36 weeks PMA) or/and death; secondary outcomes of the use of systemic corticosteroids, effect on extubation and not extubate (including extubated within 14 days and duration of mechanical ventilation), adverse outcomes of sepsis, hyperglycemia requiring treatment, intraventricular hemorrhage (IVH), periventricular leukomalacia (PVL), necrotizing enterocolitis (NEC) Bell’s stage > or = II, retinopathy of prematurity (ROP), PDA requiring drug treatment or surgery, and neurodevelopmental outcomes of neurodevelopmental impairment and cerebral palsy. Exclusion criteria were: a) non-clinical studies (experimental and basic studies); b) observational or retrospective studies; c) duplicate reports or secondary or post hoc analyses of the same study population; d) lack of sufficient information on baseline or primary or secondary outcome data; and e) therapeutic study.

### Assessment of the risk of bias

Two reviewers (Zhang and Zhong) independently assessed the risk of bias of individual studies and the bias domains across studies using the Cochrane collaboration tool [28]. All discrepancies were resolved by discussion and consensus. The studies were rated to be at high risk of bias, low risk of bias, or unclear risk of bias based on sequence generation, concealment of allocation, blinding of participants/parents and personnel, blinding of outcome assessment, incomplete outcome data, and selective outcome reporting.

### Data collection

For each study, data were extracted independently by two reviewers (Zhang and Zhong) using a predesigned form. Any differences and disagreements in the abstracted data were discussed and resolved by consensus. Details of methodological quality, study design, analysis, and results were abstracted. For each outcome, the numeric results, the statistic used, and the *P* value were abstracted. We contacted authors of the original reports to obtain further details when information regarding any of the above information was unclear.

### Statistical analysis

The statistical analyses were performed by the DerSimonian and Laird method (random-effect model) using the Review Manager meta-analysis software (version 5.3, 2012; The Cochrane Collaboration, Copenhagen, Denmark). TSA viewer version 0.9 β was used for trial sequential analysis. Treatment effect estimates for all trials were calculated, expressed as typical relative risk (RR) for dichotomous outcomes and weighted mean difference (WMD) for continuous outcomes, all with a 95% confidence interval. If the continuous measures were reported in median and inter-quartile range, mean and standard SD values were estimated using the method described by Wan et al. [29]. *P* values of ≤0.05 and RR point estimate 95% CIs that excluded the null (<1.00 or >1.00) were considered statistically significant. Where the pooled estimates of relative risk were statistically significant. We calculated the numbers needed to treat (NNT) for all outcomes. The heterogeneity between-trial regarding treatment effects was analyzed by the χ^2^ test for heterogeneity and *I* ^2^ statistics. Heterogeneity was deemed significant when the corresponding *p*-value was <0.1 or when the *I* ^2^ percentage was >50 [30]. Subgroup analyses were carried out to assess the source of heterogeneity. When more than 10 articles were included, the presence of publication bias was assessed and displayed through a funnel plot. Analysis by excluding studies at high risk of bias or abstract forms was part of a predefined subgroup analysis [28]. Sensitivity analysis with trial sequential analysis was performed to correct for random error and repetitive testing of accumulating and sparse data; meta-analysis monitoring boundaries and required information size (meta-analysis sample size) were quantified, along with D2 (diversity adjusted information size) and adjusted 95% CIs [31–34]. Risk of type 1 error was considered 5% with a power of 80%. A clinically meaningful anticipated relative risk reduction was used based on the low bias trial [16]. Trial sequential analysis 95% CI boundaries that excluded the null (<1.00 or >1.00) were considered statistically significant.

## Results

### Study selection

**Fig. 1:**
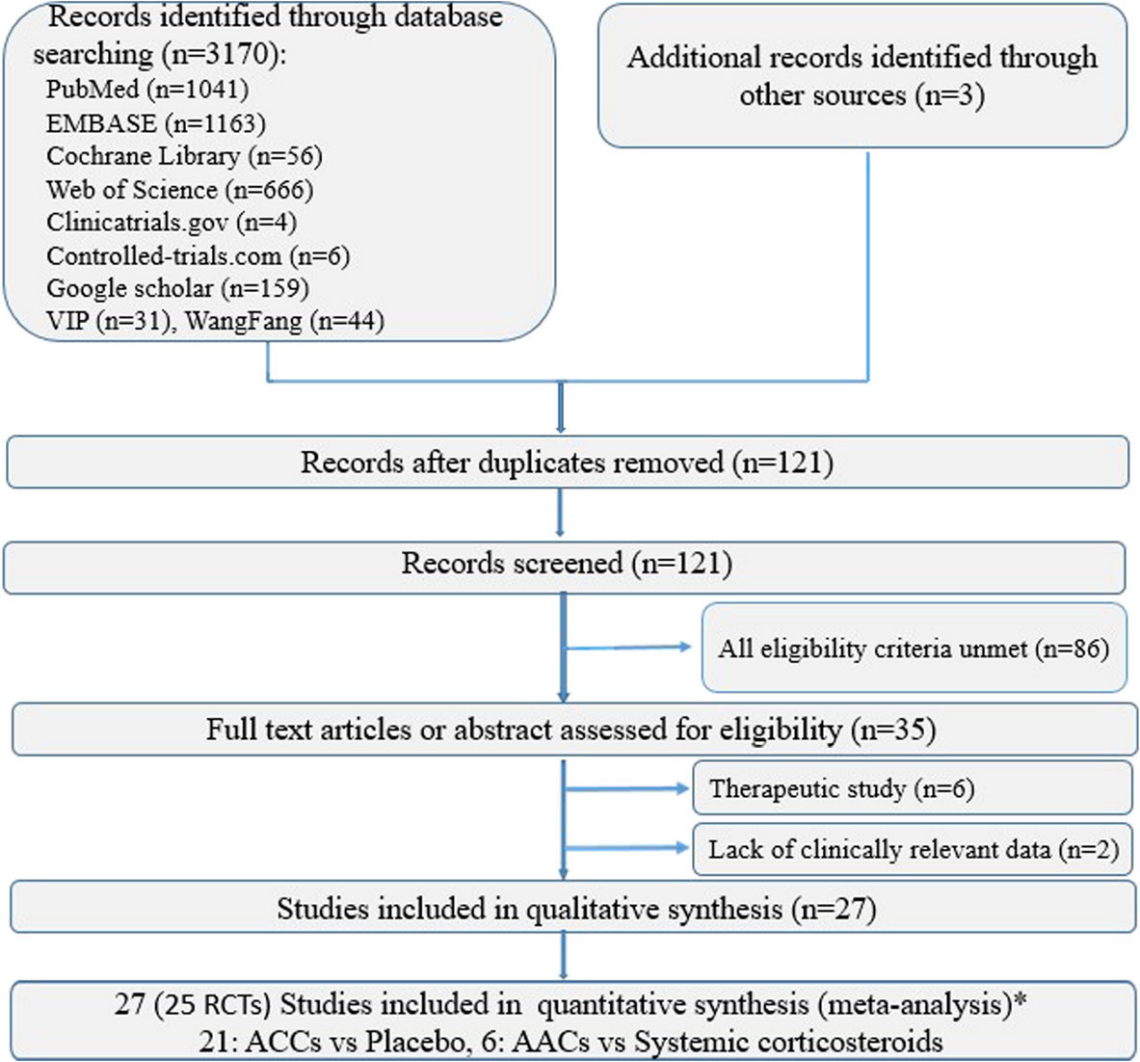
Flowchart of the study selection process. * Two article [48, 53] were follow-up study of two included trials [47, 52]

Our search identified 121 potentially relevant articles during the initial electronic database search, of which 35 RCTs were involving the use of AACs in premature infants. However, only 25 (27 articles) of 35 trials presented appropriate and sufficient data for further analysis [16, 20, 24, 25, 35–57], and the remaining eight were excluded because of therapeutic study of inhaled steroids for BPD [18, 19, 58–61] and when the raw data in Ref. [62, 63] is lacking due to unable to reach the original investigators. Overall, the 20 trials of airway administration of corticosteroid (16 trials of inhaled corticosteroid and 4 trials of instillation of steroids) vs placebo included 2484 infants, and 5 trials of inhaled corticosteroid vs systemic corticosteroids included 765 infants. Two studies were published in abstract form [20, 47]. Two articles [50, 55] were follow-up studies of two included trials [49, 54]. Fig. 1 shows the details of the selection process.

### Characteristics of the included studies

The 25 RCTs (27 articles) selected for analysis included a total of 3249 participants (Tables 1 and 2, Table S1-S2 in Additional file 1: Table S1 and Additional file 2: Table S2) [16, 20, 24, 25, 35–57]. The publication dates of the RCTs ranged from 1993 to 2016. The AACs group vs the placebo group or systemic corticosteroids group were well matched; birth weight and gestational age did not differ significantly significance. Other aspects of respiratory treatment, including the resuscitation devices used and the criteria for using antenatal glucocorticoids as well as surfactant, were adequately described in the studies and conformed to current international guidelines [64–66]. The incidence of neonatal respiratory distress syndrome (was diagnosed based on respiratory symptoms and corresponding X-ray changes) was comparable among the airway administration of corticosteroid group and comparative groups.

**Table 1:** Characteristics of 25 RCTs reported in 27 articles and baseline characteristics of patients

Study	Study characteristics	Group	N	Male (n)	GA (wk)	BW (g)	Antenatal glucocorticoid	RDS (n)	Surfactant(n)
AACs vs placebo									
Arnon [35] 1996	1 centre UK	T C	15 15	NA NA	27.5 (0.7)^a^ 27.1 (0.89)^a^	1024 (92.3)^a^ 1041 (113)^a^	7 (46.7%) 8 (53.3%)	NA NA	NA NA
Bassler [16] 2015	10 centres Switzerland	T C	437 419	NA NA	26.1 (1.3)^a^ 26.1 (1.2)^a^	798 (193)^a^ 803 (189)^a^	388 (88.8%) 383 (91.4%)	NA NA	NA NA
Cao [36] 2016	1 centre China	T C	40 40	NA NA	30.1 (2.2)^a^ 30.7 (1.8)^a^	1333 (110)^a^ 1339 (105)^a^	NA NA	40 40	40 40
Cole [37] 1999	4 centres USA	T C	123 130	51 53	26 (2)^a^ 26 (2)^a^	800 (193)^a^ 802 (189)^a^	77 (62.6%) 68 (52.3%)	123 129	95 97
Denjean [38] 1998	6 centres France	T C	43 43	26 26	27.6 (1.5)^a^ 27.8 (1.6)^a^	1060 (218)^a^ 1082 (260)^a^	11 (25.6%) 11 (25.6%)	43 43	31 34
Fok [39] 1999	1 centre Hong Kong	T C	27 26	NA NA	27.9 (0.5)^a^ 27.1 (0.5)^a^	993 (71)^a^ 981 (71)^a^	15 (55.6%) 13 (50.0%)	NA NA	NA NA
Giep [40] 1996	1 centre USA	T C	10 9	6 3	26 (2.0)^a^ 25 (1.6)^a^	752 (110)^a^ 784 (141)^a^	4 (40.0%) 2 (22.2%)	8 9	8 9
Jangaard [41] 2002	1 centre Germany	T C	30 30	13 13	27.2 (2)^a^ 27.9 (2)^a^	882 (204)^a^ 917 (178)^b^	15 (50.0%) 16 (53.3%)	19 25	19 25
Jonsson [42] 2000	1 centre Sweden	T C	15 15	8 11	25 (23,27)^b^ 26 (24,29)^b^	766 (525,1122)^b^ 813 (630,1227)^b^	12 (80.0%) 10 (66.7%)	14 15	14 15
Ke [43] 2016	1 centre China	T C	46 46	20 27	<32 <32	<1500 <1500	NA NA	46 46	46 46
LaForce [44] 1993	1 centre USA	T C	10 11	NA NA	NA NA	<1500 <1500	NA NA	NA NA	NA NA
Merz [45] 1999	1 centre Germany	T C	12 11	5 7	28 (27,32)^b^ 29 (27,31)^b^	1108 (820,1420)^b^ 1120 (880,1480)^b^	7 (58.3%) 7 (63.6%)	12 11	10 9
Nakamura [24] 2016	1 centre Japan	T C	107 104	63 50	26 (25,27)^b^ 26 (25,27)^b^	783.87 (134.8)^b^ 784.06 (127.2)^b^	45 (42.1%) 42 (40.4%)	90 88	NA NA
Pappagallo [46] 1998	1 centre USA	T C	9 9	5 5	26.8 (1.1)^a^ 26.6 (0.8)^a^	828 (64)^a^ 849 (89)^a^	NA NA	NA NA	NA NA
Townsend [47] 1998	1 centre Denver	T C	15 17	8 8	25.8 25.5	728 695	NA NA	15 17	NA NA
Wen [48] 2016	1 centre China	T C	80 80	54 51	30.5 (2.6)^a^ 31.2 (2.3)^a^	1220 (183)^a^ 1250 (167)^a^	NA NA	NA NA	NA NA
Yeh [49] 2008 Kuo [50] 2010	1 centre Taiwan	T C	60 56	31 29	26.4 (2.2)^a^ 26.7 (2.3)^a^	881 (245)^a^ 919 (272)^a^	46 (76.7%) 42 (75.0%)	60 56	60 56
Yeh [25] 2016	1 centre Taiwan	T C	131 134	NA NA	26.5 (2.2)^a^ 26.8 (2.2)^a^	882 (249)^a^ 935 (283)^a^	NA NA	131 134	131 134
Yong [20] 1999	1 centre UK	T C	20 20	13 12	27.4 (1.7)^a^ 27.7 (1.7)^a^	1011 (223)^a^ 932 (401)^a^	NA NA	NA NA	NA NA
Zimmerman [51] 2000	1 centre USA	T C	20 19	12 8	26 (2.0)^a^ 26 (2.0)^a^	910 (198)^a^ 802 (225)^a^	14 (70.0%) 15 (78.9%)	15 18	15 18
Inhaled corticosteroids vs systemic corticosteroids									
Dimitriou [52] 1997	1 centre UK	T C	20 20	NA NA	27 (24,30)^b^ 27 (24,31)	849 (584,1270)^b^ 818 (425,1460)^b^	12 (60.0%) 11 (55.0%)	NA NA	14 12
Groneck [53] 1999	1 centre Germany	T C	7 9	3 3	26 (25,28)^b^ 26 (25,28)^b^	800 (500,1020)^b^ 847 (660,1030)^b^	NA NA	NA NA	NA NA
Halliday [54] 2001 Wilson [55] 2006	47 centre Europe	T C	285 285	142 164	27.2 (1.9)^a^ 27.2 (1.9)^a^	1002 (281)^a^ 1011 (285)^a^	177 (62.1%) 164 (57.5%)	NA NA	265 260
Rozycki [56] 2003	1 centre USA	T C	46 15	23 7	<31 <31	<1500 <1500	6 (13.0%) 2 (13.3%)	46 15	46 15
Suchomski [57] 2002	1 centre USA	T C	51 27	25 19	26 (1.6)^a^ 26 (2.0)^a^	844 (157)^a^ 843 (227)^a^	34 (66.7%) 16 (59.3%)	NA NA	NA NA

*RCTs* Randomized controlled trials, *AACs* Airway administration of corticosteroids, *RDS* Respiratory distress syndrome, *PS* Pulmonary surfactant, *AG* Antenatal glucocorticoid, *GA* Gestational age, *BW* Brith weight, ^a^means ± SD, ^b^median (25th, 75th percentiles), NA: Not Applicated

**Table 2:** Interventions used in the 25 RCTs

Study	Age at enrollment	Type of AACS	Intervention dose	Mode of delivery	Intervention treatment
AACs vs placebo					
Arnon [35] 1996	15 (0.6)^a^ 14 (0.5)^a^	BUD	600 μg BID	MDI	7 days or until the infant had been extubated
Bassler [16] 2015	within 12 h	BUD	400 μg Q12H for 14 days 200 μg Q12H for 15 days	MDI	Until extubation and no oxygen dependent or 32 weeks PMA
Cao [36] 2016	within 6 h	BUD	250 μg Q8H	Airway instillation	Until FIO_2_ < 0.4 or extubation
Cole [37] 1999	5.7 (3.4)^a^ 5.4 (2.9)^a^	BDP	40 μg/kg for 7 days 30 μg/kg for 7 days 15 μg/kg for 7 days 5-10 μg/kg for 7 days	MDI	28 days
Denjean [38] 1998	10 days	BDP	250 μg Q6H	MDI	28 days
Fok [39] 1999	within 24 h of birth	FPP	250 μg Q12H	MDI	14 days
Giep [40] 1996	14 days	BDP	1 mg/kg/d	MDI	7 days
Jangaard [41] 2002	3 years	BDP	0.2 mg/kg/d	MDI	Until 28 days of age
Jonsson [42] 2000	6 days	BUD	0.5 mg/times BID	Electronic dosimetric jet nebulizer	14 days
Ke [43] 2016	within 6 h	BUD	250 μg Q12H	Airway instillation	Until stop respiratory support
LaForce [44] 1993	14 days	BDP	50 μg Q8H	A Whisper Jet nebulizer system	28 days
Merz [45] 1999	3 days	BUD	1.6 mg/d Q6H	MDI	10 days or until extubation
Nakamura [24] 2016	within 24 h of birth	FPP	50 μg BID	MDI	Until 6 weeks of age or extubation
Pappagallo [46] 1998	22.6 (3.0)^a^ 19.1 (1.6)^a^	DXM	1 mg/kg Q8H for 7 days 0.5 mg/kg Q8H for 7 days	A jet nebulizer	10 days
Townsend [47] 1998	at 48-96 h of age	FPP	500 μg TID	MDI	28 days or until extubation
Wen [48] 2016	within 24 h of birth	BUD	0.4 mg/kg Q12H	MDI	14 days
Yeh [49] 2008 Kuo [50] 2010	2.1 (2.2)^a^	BUD	0.25 mg/kg Q8H	Airway instillation	Until the infant required ≤0.4 of FIO2 or until the infant was extubated
Yeh [25] 2016	2.0 (1.5)^a^ 1.8 (1.6)^a^	BUD	0.25 mg/kg Q8H	Airway instillation	Until the infant required ≤0.4 of FIO_2_ or until extubation
Yong [20] 1999	within 18 h of birth	FPP	250 μg Q12H	MDI	14 days
Zimmerman [51] 2000	within 24 h of birth	BDP	2MDI, Q6H for 3 days; 2MDI, Q8H for 3 days; 2MDI Q12H for 3 days; 2MDI QD for 3 days	MDI	12 days
Inhaled corticosteroids vs systemic corticosteroids					
Dimitriou [52] 1997	After 5 days	BUD	100 μg QD	A jet nebulizer	10 days
Groneck [53] 1999	After 72 h	BDP	250 μg QD	MDI	10~28 days
Halliday [54] 2001 Wilson [55] 2006	< 72 h or > 15d	BUD	<1000 g 0.4 mg BID >1000 g 0.6 mg BID	MDI	12 days or until extubation
Rozycki [56] 2003	at 14 days of age	BDP	High: 2.4-3.7 mg/kg/d Mid: 1.0-1.8 mg/kg/d Low: 0.5-0.7 mg/kg/d	MDI	7 days or until extubation
Suchomski [57] 2002	12-21 days of age	BDP	High: 800 μg/d Low: 400 μg/d	MDI	Until extubation

*RCTs* Randomized controlled trials, *BUD* Budesonide, *BDP* Beclomethasone dipropionate, *FPP* Fluticasone propionate, *MDI* metered-dose inhaler, *AACs* Airway administration of corticosteroids, *DXM* Dexamethasone

None of the trials were sponsored by the manufacturer

^a^means ± SD

Although all studies attempted to include infants at risk of developing BPD, the inclusion criteria, the intervention (type of AACs), and duration of therapy varied between studies. The intervention regimens,including the prescribed dosages and administration schedules, varied considerably between the RCTs. Overall, the duration of AACs treatment ranged from 3 to 28 days, and the types of AACs included beclomethasone, budesonide, fluticasone, and dexamethasone. Delivery systems included metered dose inhaler (MDI) with a spacer device, nebulization, and airway instillation. Four studies used airway instillation of budesonide using surfactant as a vehicle [25, 36, 48, 49].

### Risk of bias within individual studies

**Fig. 2:**
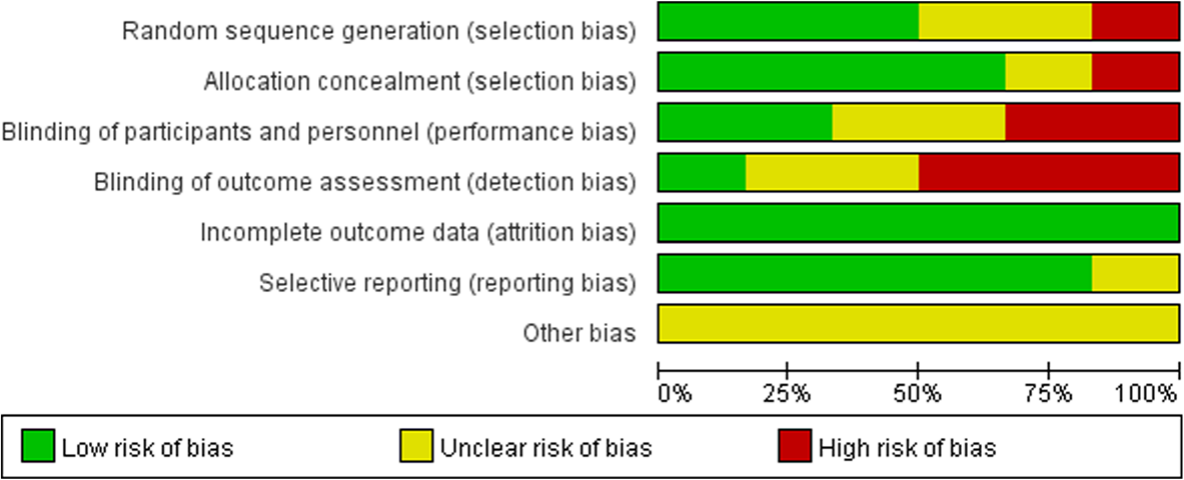
Risk of bias graph

**Fig. 3:**
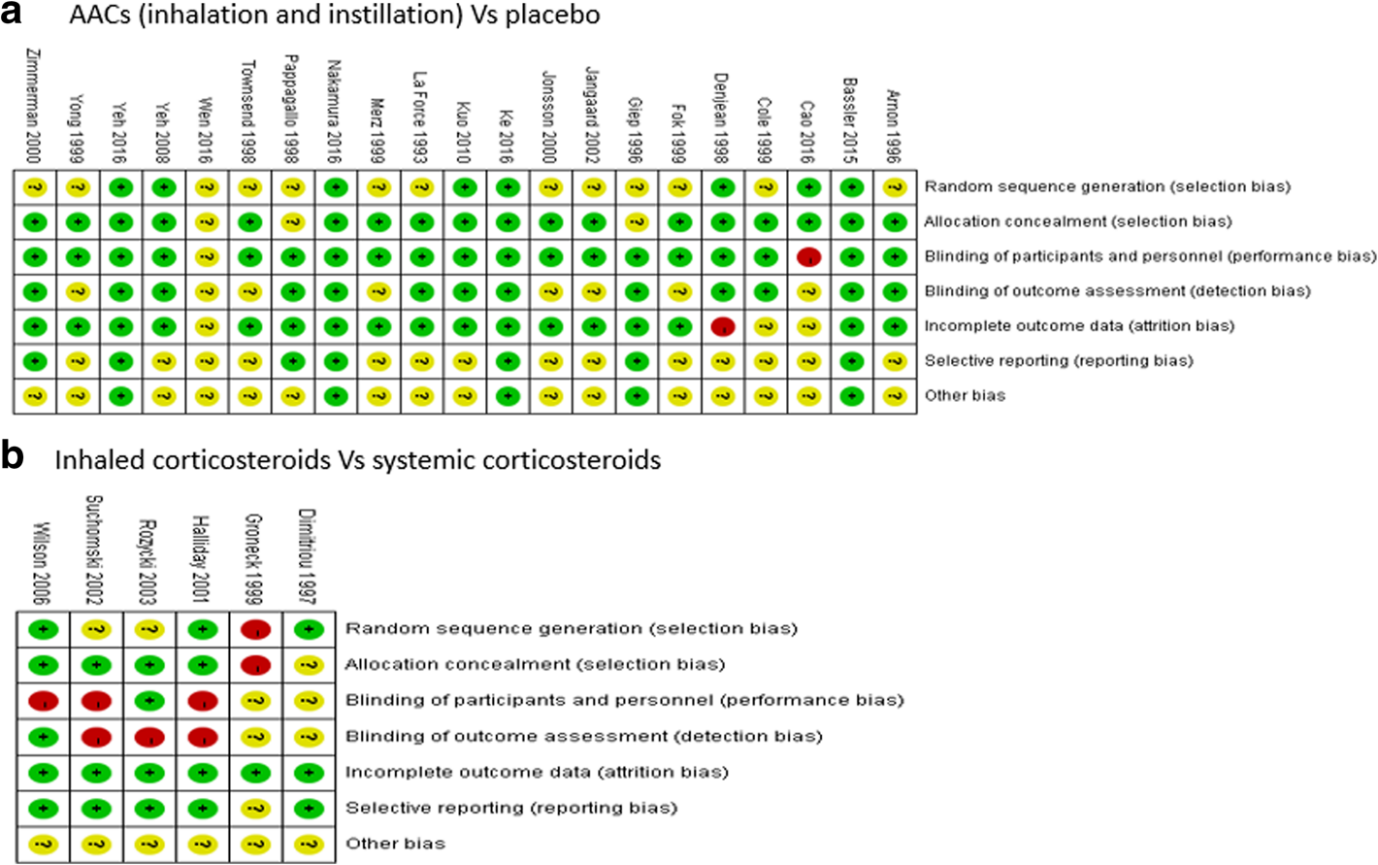
Risk of bias summary for all included records

Three studies were deemed to have a low risk of bias [16, 24, 25], seven studies were deemed to have a high risk of bias [36, 38, 53–57] due to selection bias, detection bias, attrition bias or performance bias, and 17 studies were classified as having an unclear risk of bias [20, 35, 37, 39–52]. Although all studies were presented as randomized trials, the method of randomization was determined to be inadequate in 17 studies [20, 35, 37, 39–48, 51, 53, 56, 57]. Sixteen studies were found to have adequate concealment of allocation and clearly described blinding for the intervention method [16, 20, 24, 25, 38, 39, 41, 42, 44, 45, 47, 49–51, 56]. Thirteen studies were found to have adequate blinding of outcome assessment [16, 24, 25, 35, 37, 38, 40, 44, 46, 49, 50, 52, 56]. Follow-up data were reported in five studies [24, 25, 41, 50, 56]. Fig. 2 and 3 summarise the risks of bias.

### Systematic review of the findings from synthesis of the results

## ACCs vs placebo

### Primary clinical outcome

#### BPD

**Fig. 4:**
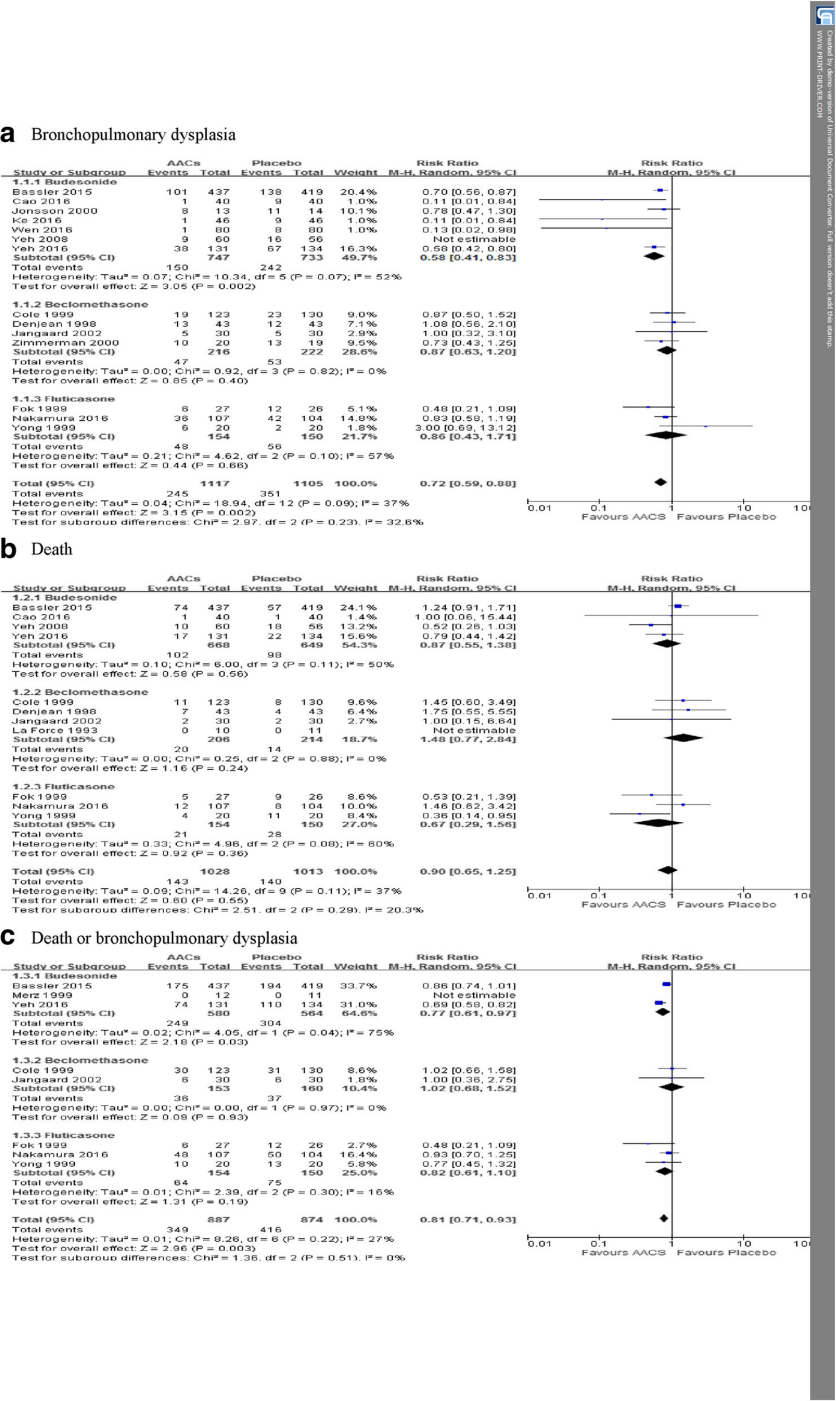
Meta-analysis of primary outcome with the use of AACs or placebo, AACs: Airway administration of corticosteroids

**Fig. 5:**
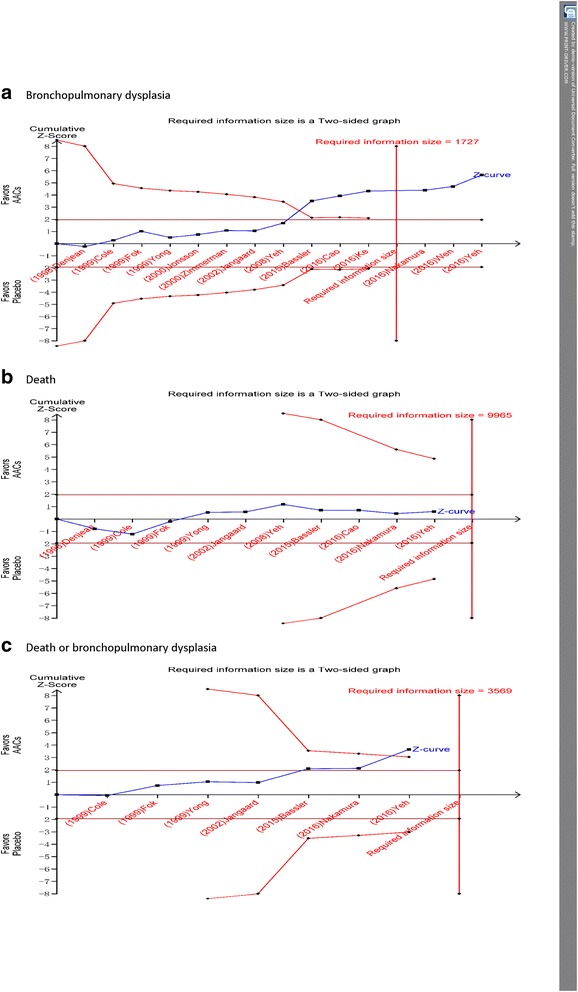
Trial sequential analysis of trials reporting primary outcome comparing pooled AACs and placebo. A diversity adjusted information size was calculated using ɑ = 0.05 (two sided), β = 0.20 (power 80%), D2 = 0%, an anticipated relative risk of 29.8%, −24.5%, 13.5%, respectively, and an event proportion of 28.2%, 11.4%, 46.3%, respectively in the control arm. The cumulative z curve was constructed using a random-effect model, and a penalized z curve was also constructed. The adjusted relative risk was 0.71, 0.90, 0.81, respectively, and the 95% confidence interval was corrected to 0.57–0.87, 0.40–2.03, and 0.71–0.97, respectively, AACs: Airway administration of corticosteroids

Fourteen trials enrolling 2388 neonates reported on the incidence of BPD. Meta-analysis indicated that AACs was associated with a lower likelihood of BPD than was placebo (RR = 0.71, 95% CI 0.58 to 0.86, NNT = 10, *I* ^2^ = 34%, *P* = 0.0005) (Fig. 4). Trial sequential analysis correction of the 95% confidence interval (0.57 to 0.87; D^2^ = 53%) did not alter the finding of BPD morbidity benefit with AACs (Fig.5). The finding was robust to sensitivity analysis (highest *P* value 0.007) and the subgroup of instillation of steroids had the lowest NNT (Table 3), and clear evidence of publication bias was not present (Figure S1 in Additional file 3). Subgroup analysis based on type of corticosteroid showed that the incidence of BPD was significantly lower only in the group treated with budesonide compared to placebo (RR = 0.59, 95% CI 0.44 to 0.79, NNT = 8, *I* ^2^ = 40%, *P* = 0.0004) (Fig. 4). In the trial sequential analysis, the cumulative z score crossed the sequential monitoring boundary of benefit and the required information size (Fig. 5), suggesting that further studies have little chance to perturbation the finding of benefit from AACs.

**Table 3:** Sensitivity analysis of primary outcomes with the use of AACs or placebo

Outcome or Subgroup	Studies	Participants	P value	Effect Estimate	NNT
BPD	14	2338	0.0005	0.71 [0.58, 0.86]	10
Exclusion of high risk trials	12	2172	0.0001	0.70 [0.58, 0.84]	10
Exclusion of conference abstracts	13	2298	<0.0001	0.69 [0.58, 0.83]	9
Exclusion of non English studies	11	2006	<0.00001	0.72 [0.63, 0.83]	10
Exclusion of small sample trials	6	1861	<0.0001	0.69 [0.57, 0.83]	10
Inhaled corticosteroids	10	1785	0.003	0.77 [0.65, 0.91]	14
Instillation of steroids using surfactants as carriers	4	553	0.007	0.44 [0.24, 0.79]	5
Death	11	2041	0.55	0.75 [0.54, 1.04]	
Exclusion of high risk trials	9	1875	0.40	0.88 [0.56, 1.39]	
Exclusion of conference abstracts	10	2003	0.99	1.00 [0.75, 1.33]	
Exclusion of non English studies	10	1961	0.55	0.90 [0.64, 1.27]	
Exclusion of small sample trials	5	1701	0.98	1.01 [0.70, 1.45]	
Inhaled corticosteroids	8	1580	0.88	1.03 [0.69, 1.53]	
Instillation of steroids using surfactants as carriers	3	461	0.07	0.67 [0.43, 1.04]	
Death or BPD	8	1761	0.003	0.81 [0.71, 0.93]	12
Exclusion of conference abstracts	7	1721	0.01	0.82 [0.70, 0.96]	12
Exclusion of small sample trials	4	1585	0.03	0.83 [0.70, 0.99]	13
Inhaled corticosteroids	7	1496	0.03	0.87 [0.77, 0.99]	20
Instillation of steroids using surfactants as carriers	1	265	<0.0001	0.69 [0.58, 0.82]	4

*AACs* Airway administration of corticosteroids, *BPD* Bronchopulmonary dysplasia, *NNT* Numbers needed to treat

#### Death

Eleven trials enrolling 2041 neonates reported on the incidence of death. Meta-analysis indicated that the incidence of death was not significantly different between the AACs group and the placebo group (RR = 0.90, 95% CI 0.65 to 1.25, *I* ^2^ = 37%, *P* = 0.55) (Fig. 4). Trial sequential analysis correction of the 95% confidence interval (0.40 to 2.03; D^2^ = 55%) did demonstrate no increasing risk of death with airway administration of corticosteroid (Fig.5). The finding was robust to sensitivity analysis (Table 3), and clear evidence of publication bias was not present (Figure S1 in Additional file 3). Sensitivity analysis showed that instillation of steroids using surfactant as a vehicle had a nonsignificant reduction mortality (12.1% vs. 17.8%, RR = 0.67; 95% CI 0.43 to 1.04, *I* ^2^ = 0%, *P* = 0.07) (Table 3).

#### Death or BPD

Eight trials enrolling 1761 neonates reported on the incidence of death or BPD. Meta-analysis indicated that airway administration of corticosteroid was associated with a lower likelihood of death or BPD than was placebo (RR = 0.81, 95% CI 0.71 to 0.93, NNT = 12, *I* ^2^ = 27%, *P* = 0.003) (Fig. 4). Trial sequential analysis correction of the 95% confidence interval (0.71 to 0.97; D^2^ = 55%) did support the finding of death or BPD morbidity benefit with airway administration of corticosteroid (Fig.5). The finding was robust to sensitivity analysis (highest *P* value 0.03) (Table 3). Subgroup analysis based on type of corticosteroid showed that the incidence of death or BPD was significantly lower only in the group treated with budesonide versus placebo (RR = 0.77, 95% CI 0.61 to 0.97, NNT = 9, *I* ^2^ = 75%, *P* = 0.03) (Fig. 4).

### Secondary outcomes

#### Requirement for systemic steroids

Fourteen trials enrolling 1886 neonates reported on the requirement for systemic steroids. A significant reduction in the administration of systemic corticosteroids was found in the group with airway administration of corticosteroids (RR = 0.86, 95% CI 0.76 to 0.97, NNT = 20 *I* ^2^ = 1%, *P* = 0.02). However, publication bias was present (Figure S1 in Additional file 3). Subgroup analysis based on type of corticosteroid showed that the requirement for systemic steroids was significantly different only with beclomethasone versus placebo (Figure S2 in Additional file 4).

#### Benefit to extubation

Seven trials enrolling 552 neonates reported on rates of successful extubation within 14 days. AACs have a nonsignificant increasing rates of successful extubation within 14 days (55.1% vs. 44.6%, RR = 1.53, 95% CI 1.0 to 2.33, *I* ^2^ = 66%, *P* = 0.05) (Figure S3 in Additional file 5). Seven trials enrolling 623 neonates reported on the the duration of mechanical ventilation. AACs significantly reduce the duration of mechanical ventilation compared with placebo (WMD = −2.99, 95% CI -5.10 to −0.87, *I* ^2^ = 38%, *P* = 0.006) (Figure S4 in Additional file 6).

#### Adverse outcomes

AACs administration had a nonsignificant reduction in incidence of PDA (40.2% vs. 45.2%, RR = 0.89, 95% CI 0.79 to 1.0, *I* ^2^ = 0%, *P* = 0.05) and NEC (7.3% vs. 9.9%, RR = 0.75, 95% CI 0.54 to 1.04, *I* ^2^ = 0%, *P* = 0.09) compared with placebo (Table S3 in Additional file 7). There were no significant differences between interventions in the likelihood of other adverse outcomes (including infection, hyperglycaemia, IVH, PVL, and ROP) (Table S3 in Additional file 7).

#### Neurodevelopmental outcomes

Four trials enrolling 474 neonates reported on the neurodevelopmental outcomes (neurodevelopmental impairment and cerebral palsy). Meta-analysis indicated that the incidence of neurodevelopmental impairment and cerebral palsy ware not significantly different between the airway administration of corticosteroid group and the placebo group (Table S3 in Additional file 7).

## Inhaled corticosteroids vs systemic corticosteroid

### Primary clinical outcome

#### Bpd

**Fig. 6:**
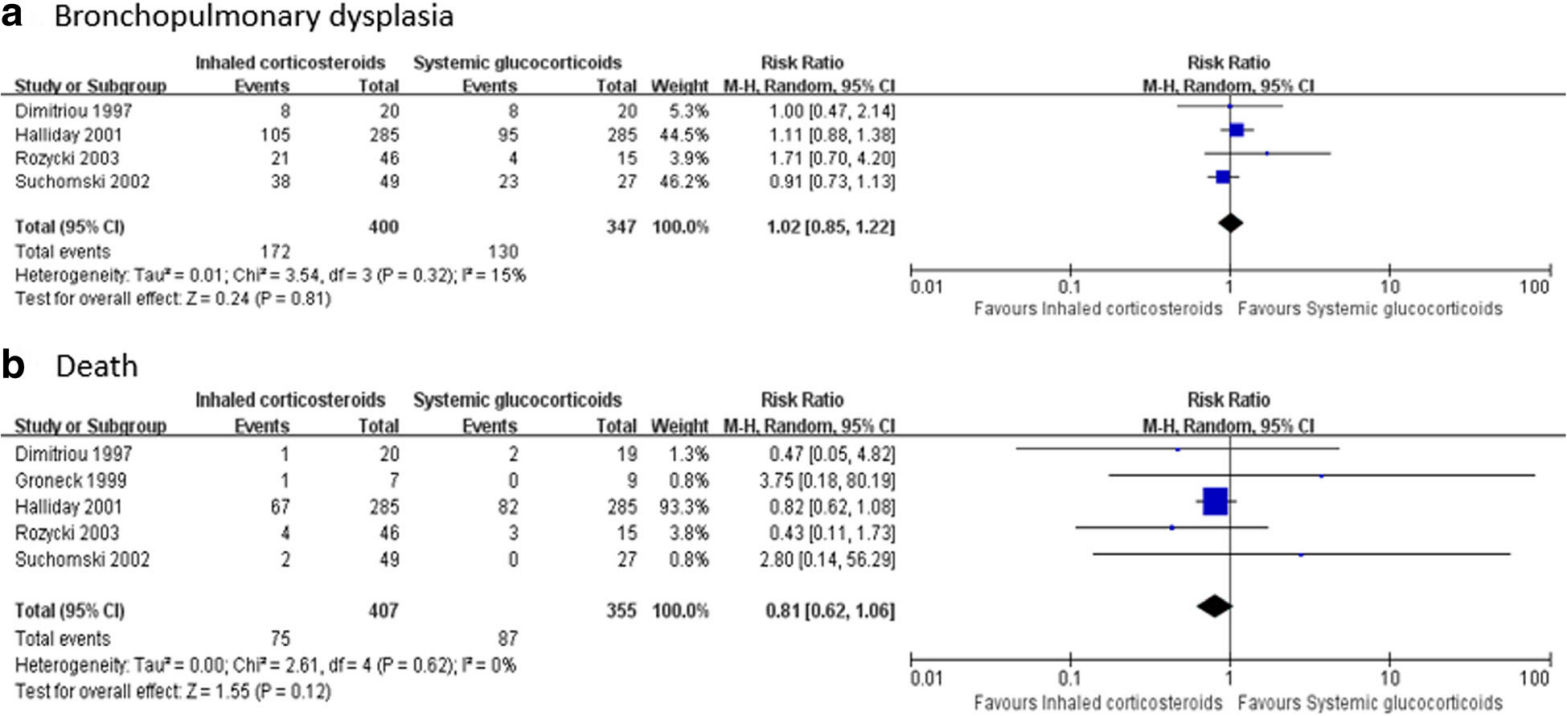
Meta-analysis of primary outcome with the use of inhaled corticosteroids or systemic corticosteroids

**Fig. 7:**
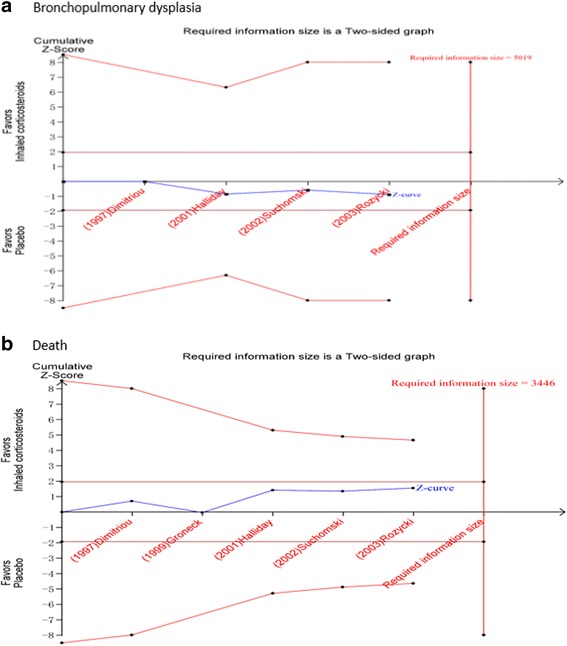
Trial sequential analysis of trials reporting primary outcome comparing pooled inhaled corticosteroids and systemic corticosteroids. A diversity adjusted information size was calculated using ɑ = 0.05 (two sided), β = 0.20 (power 80%), D2 = 0%, an anticipated relative risk of −10.5%, 18.4%, respectively, and an event proportion of 36.6%, 20.0%, respectively in the control arm. The cumulative z curve was constructed using a random-effect model, and a penalized z curve was also constructed. The adjusted relative risk was 1.08, 0.81, respectively, and the 95% confidence interval was corrected to 0.53–2.20, 0.43–1.53, respectively

Four trials enrolling 747 neonates reported on the incidence of BPD. Meta-analysis indicated that the morbidity of BPD has no decisive difference between inhaled corticosteroid group and systemic corticosteroid group (RR = 1.02, 95% CI 0.85 to 1.22, *I* ^2^ = 15%, *P* = 0.81) (Fig. 6). The finding was robust to trial sequential analysis (RR = 1.02, adjusted 95% CI 0.55 to 1.88, *D* ^2^ = 0%, *P* = 0.83) (Fig. 7).

#### Death

Five trials enrolling 762 neonates reported on the incidence of death. Meta-analysis indicated that the mortality was not significantly different between the inhaled corticosteroid group and the systemic corticosteroid group (RR = 0.81, 95% CI 0.62 to 1.06, *I* ^2^ = 0%, *P* = 0.12) (Fig. 6), and this finding was confirmed in trial sequential analysis (adjusted 95% confidence interval 0.43 to 1.53). The cumulative Z curve did not cross any boundaries for benefit or harm (Fig. 7).

### Secondary outcomes

#### Benefit to extubation

Four trials enrolling 693 neonates reported on the the duration of mechanical ventilation. Systemic corticosteroids were associated with shorter duration of mechanical ventilation (WMD = 3.21, 95% CI 0.36 to 6.06, *I* ^2^ = 10%, *P* = 0.03) (Figure S5 in Additional file 8).

#### Adverse outcomes

Inhaled corticosteroids were associated with less hyperglycemia (RR = 0.44, 95% CI 0.29 to 0.69, NNT = 9, *I* ^2^ = 0%, *P* = 0.0003) (Table S4 in Additional file 1). There were no significant differences between interventions in the likelihood of other adverse outcomes (including infection, NEC, PDA, PVL, and ROP) (Table S4 in Additional file 9).

#### Neurodevelopmental outcomes

One trial enrolling 126 neonates reported on the neurodevelopmental outcomes (impairment, disability, and cerebral palsy). There were no significant differences between interventions (Table S4 in Additional file 9).

## Discussion

This meta-analysis including 25 trials with 3249 preterm infants at high risk of BPD estimated relative effects of AACs. The use of AACs was associated with a lower likelihood of the primary outcomes of BPD and death or BPD, and secondary outcomes of requirement for systemic steroids and the duration of mechanical ventilation than placebo, and was equivalent for preventing BPD than systemic corticosteroids. Moreover, AACs were not associated with an increased risk of death compared with placebo or systemic corticosteroids. AACs had less occurrence of hyperglycemia compared to systemic steroids.

### Interpretation of the findings

Our primary outcomes, were robust to sensitivity and trial sequential analyses. When evaluating the incidence of BPD, death, and death or BPD between AACs and placebo, consistent results was observed in trial sequential analyses and after exclusion of studies at high risk of bias, small sample, non-English literature, abstract, or studies of instillation of steroids using surfactants as carriers. In addition, sensitivity analysis showed that NNT of 5 and 4 in subgroup of instillation of steroids using surfactant as a vehicle was lower than the NNT in the inhaled corticosteroids group (NNT = 14 and 20) for preventing one case of BPD and BPD or death (Table 3). Using as a vehicle surfactant may also enhance the solubility of budesonide, increase budesonide absorption, and strengthen the function of anti-inflammatory effects [25, 67]. Trial sequential analysis corrected the 95% CIs of the already statistically significant point estimates for main comparison of an airway administration of corticosteroid with the placebo through accounting for random error and repetitive testing of accumulating sparse data. In our analysis of incidence of BPD and death or BPD between AACs and placebo, the cumulative z score entered the futility area, indicating further trials are not required. Considering the overall morbidity, no association with benefit or harm can be found between groups, but the trial sequential analysis suggested it would be unnecessary to carry out more trials on this outcome. In our analysis of neurodevelopmental outcome between AACs and placebo, AACs did not increase neurodevelopmental impairment. Futhermore, inhaled corticosteroid also did not increase mortality and is similar in preventing BPD compared with systemic corticosteroids. The information size for the comparison of inhaled corticosteroids with systemic corticosteroids was too low to require futility boundaries in trial sequential analysis. European Consensus Guidelines on the Management of Respiratory Distress Syndrome do not recommend the use of inhaled corticosteroid for preventing BPD until further safety data become available [68]. The benefit of preventing BPD and death or BPD associated with AACs found in this systematic review could inform future updates of these and other clinical guidelines.

### Comparison with other studies

Several systematic reviews have evaluated inhaled corticosteroids in the prevention of BPD [14, 15, 17, 21, 22]. A recent meta-analysis aimed to assess inhaled corticosteroids for BPD in preterm infants [17]. The evidence from this meta-analysis supported inhaled corticosteroids, especially budesonide, as a potentially efficacious and safe therapy for the prevention or treatment of BPD in preterm infants [17]. However they fail to implement sensitivity and subgroup analysis for the result of heterogeneity. In addition, they did not evaluate the advantage on inhaled corticosteroids compared with sysetmic corticosteroids. The previous Cochrane reviews examined either inhalation or systemic corticosteroids, further divided into early and late phases according to the time of administration. Hence, the analysis used 1 week after birth as a boundary [21, 22] However, in our meta-analysis, the duration of inhalation of corticosteroids ranged from 3 to 29 days, which was different from studies on intravenous administration, for which the course was about 1 week. For a long course of AACs, the boundary of 1 week after birth is sufficient to reflect the differences of AACs. Studies set the first delivery time from 12 h to 14 days after birth and the time span was long (Table 1). Therefore, we hypothesize that the subgroup analysis with 1 week after birth as the boundary is not reliable, and it is difficult to follow. The dose and duration of the AACs in each study were not the same, making it difficult to determine the exact dose and the course of corticosteroids that is supposed to be beneficial even via subgroup analysis. We believe that a unified drug delivery time and course of treatment are very important for developing precise treatment and firm recommendations.

### Strengths and limitations of this review

The results of our analysis confirm those of the previous systematic reviews while improving their precision and further reducing the role of contingency. A major strength of present systematic review is the use of the robust Cochrane methodology [26, 28, 30], and the meta-analysis is further challenged with trial sequential analysis to correct for random error and repetitive testing, which often is biased towards an intervention [31–33]. Our meta-analysis did focuse on bias and quality of evidence of included studies in accordance with GRADE [69]. Predefined sensitivity, subgroup analysis, and trial sequential analyses that included assessment of bias and clinically heterogenous groups were presented to aid healthcare professionals for clinical decisions. However, subtle underlying bias of the trials included in the review remains a possible limitation, as in any other systematic review although we excluded the studies that are at high risk of bias.

Admittedly, several limitations in our meta-analysis might have affected the interpretation of findings. First, the trials analyzed differed in their study design and clinical characteristics of the study participants. The effect on mortality and BPD might be quite different between very preterm infants (GA: 28~32 weeks) and extremely preterm infants (GA < 28 weeks), and subgroup analysis based on GA or BW could not be done due to the lack of individual patient data. For extremely preterm infants, the BPD definition is based on respiratory support and oxygen requirements at 36 weeks, which may be dependent on local practices and saturation targets. Despite attempts to standardize the definition of BPD, wide variation among centers has been reported with the diagnosis of BPD ranging from 6 to 57% depending on the definition chosen [70]. Hence the target population in each studies may be very different. The duration, dose, type, and inhalation or instillation of steroids using surfactants as carriers also were inconsistent across studies. Although we validated the stability of the result of our meta-analysis by subgroup analysis and sensitivity, the direct comparison between inhalation and instillation steroids using surfactant as a vehicle was lacking. Furthermore, there were few studies on AACs compared with systemic corticosteroids, and not all trials presented the primary and secondary outcomes, be especially neurodevelopmental outcomes. To some extent, the clinical diversity still potentially compromises the validity of the current results, even though we have pooled the data as if they were from clinically homogeneous studies. Third, formal tests for publication bias are still lacking. In principle, this should not interfere with the meta-analysis results, because publication bias generally results in an overestimation of the effect estimates.

## Conclusions

In this meta-analysis with sequential analysis, compared with placebo, AACs (especially instillation of budesonide using surfactant as a vehicle) was found to reduce the risk of BPD and death or BPD, with no assocaited increase in mortality and neurodevelopmental impairment. Compared with systemic corticosteroids, inhaled corticosteroids were similar for preventing BPD. The appropriate dose and duration, inhalation or instillation with surfactant as a vehicle and the long-term safety of AACs needs to be assessed in large trials.

## Additional files

Additional file 1: Table S1. Outcomes measured in the 25 RCTs (DOCX 27 kb)

Additional file 2: Table S2. Outcomes measured in the 25 RCTs (DOCX 29 kb)

Additional file 3: Figure S1. Funnel plot of bronchopulmonary dysplasia, death, and requirement for systemic steroids with the use of AACs or placebo, AACs: Airway administration of corticosteroids. (JPEG 50 kb)

Additional file 4: Figure S2. Meta-analysis of requirement for systemic steroids with the use of AACs or placebo, AACs: Airway administration of corticosteroids. (JPEG 26 kb)

Additional file 5: Figure S3. Meta-analysis of success to extubate within 14 days with the use of AACs or placebo, AACs: Airway administration of corticosteroids. (JPEG 20 kb)

Additional file 6: Figure S4. Meta-analysis of duration of mechanical ventilation with the use of AACs or placebo, AACs: Airway administration of corticosteroids. (JPEG 17 kb)

Additional file 7: Table S3. Subgroup analysis of adverse and neurodevelopmental outcomes with the use of AACs or placebo (DOCX 17 kb)

Additional file 8: Figure S5. Meta-analysis of duration of mechanical ventilation with the use of inhaled corticosteroids or systemic corticosteroids. (JPEG 11 kb)

Additional file 9: Table S4. Subgroup analysis of adverse and neurodevelopmental outcomes with the use of inhaled corticosteroids or systemic corticosteroids. (DOCX 15 kb)

## Abbreviations

AACs: Airway administration of corticosteroids
BPD: Bronchopulmonary dysplasia
IVH: Intraventricular hemorrhage
MDI: Metered dose inhaler
NEC: Necrotizing enterocolitis
NNT: Number needed to treat
PDA: Patent ductus arteriosus
PMA: Postmenstrual age
PVL: Periventricular leukomalacia
RCTs: Randomized controlled trials
ROP: Retinopathy of prematurity
RR: Relative risk
WMD: Weighted mean difference
