# Supplementary material for: Airway administration of corticosteroids for prevention of bronchopulmonary dysplasia in premature infants: a meta-analysis with trial sequential analysis
Source: BMC Pulm Med. 2017 Dec 15;17:207. doi: 10.1186/s12890-017-0550-z (PMC5732371; doi:10.1186/s12890-017-0550-z)
Supplement: Supplementary file 1 — Outcomes measured in the 25 RCTs (DOCX 27 kb) [file 12890_2017_550_MOESM1_ESM.docx]

**Additional file 1: Table S1 Outcomes measured in the 25 RCTs**

| **Study** | **Group** | **BPD** | **Death** | **Death or BPD** | **Requirement for systemic steroids** | **Extubate within 14 days** | | **Mechanical ventilation, days** |
| --- | --- | --- | --- | --- | --- | --- | --- | --- |
| **AACs vs placebo** | | | | | | |  |  |
| Arnon^35^  1996 | T  C |  |  |  |  |  | |  |
| Bassler^16^  2015 | T  C | 101  138 | 74  57 | 175  194 | 127  134 |  | |  |
| Cao^36^  2016 | T  C | 1  9 | 1  1 |  |  |  | | 4.1 (1.0)*  6.4(1.1)* |
| Cole^37^  1999 | T  C | 19  23 | 11  8 | 30  31 | 44  62 |  | | 26 (15, 43)**  37 (17, 48)** |
| Denjean^38^  1998 | T  C | 13  12 | 7  4 |  | 16  15 |  | | 22.1 (17.1)*  24 (13.9)* |
| Fok^39^  1999 | T  C | 6  12 | 5  9 | 6  12 | 5  8 | 17  8 | |  |
| Giep^40^  1996 | T  C |  |  |  | 2  3 |  | |  |
| Jangaard^41^  2002 | T  C | 5  5 | 2  2 | 6  6 | 14  21 | 12  15 | |  |
| Jonsson^42^  2000 | T  C | 8  11 |  |  | 1  3 | 7  2 | | 11 (1–40)***  14 (1–38)*** |
| Ke^43^  2016 | T  C | 1  9 |  |  |  |  | |  |
| LaForce^44^  1993 | T  C |  | 0  0 |  |  |  | |  |
| Merz^45^  1999 | T  C |  |  | 0  0 | 7  6 | 5  4 | | 14.5 (5–19)***  15 (7–20)*** |
| Nakamura^24^  2016 | T  C | 36  42 | 12  8 | 48  50 |  |  | |  |
| Pappagallo^46^ 1998 | T  C |  |  |  | 2  4 |  | |  |
| Townsend^47^  1998 | T  C |  |  |  | 13  15 |  | |  |
| Wen^48^  2016 | T  C | 1  8 |  |  |  |  | |  |
| Yeh^49^ 2008  Kuo^50^ 2010 | T  C | 9  16 | 10  18 |  | 5  7 | 27  9 | | 14.6 (19.2)*  19.5 (23.5)* |
| Yeh^25^  2016 | T  C | 38  67 | 17  22 | 74  110 | 4  9 | 80  80 | |  |
| Yong^20^  1999 | T  C | 6  2 | 4  11 | 10  13 | 9  1 | 7  6 | |  |
| Zimmerman^51^ 2000 | T  C | 10  13 |  |  | 12  14 | 8  2 | | 20 (16)*  37 (19)* |
| **Inhaled corticosteroids vs systemic corticosteroids** | | | | | | |  |  |
| Dimitriou^52^  1997 | T  C | 8  8 | 1  2 |  |  |  | | 10 (1–35)***  19 (3–57)*** |
| Groneck^53^  1999 | T  C |  | 1  0 |  |  |  | | 21(7)*  15.2(1.2)* |
| Halliday^54^ 2001  Wilson^55^2006 | T  C | 105  95 | 75  87 |  |  |  | | 11 (6–29),13 (7–31)**  9 (5–20), 13 (6–27)** |
| Rozycki^56^  2003 | T  C | 21  4 | 4  3 |  |  |  | |  |
| Suchomski^57^ 2002 | T  C | 38  23 | 2  0 |  |  |  | | 50 (21)*  53 (32)* |

**T: Treatment, C: Control, AACs: Airway administration of corticosteroids, BPD: Bronchopulmonary dysplasia, *means ±SD, **median (25th, 75th percentiles), ***median (range)**
