# Supplementary material for: Airway administration of corticosteroids for prevention of bronchopulmonary dysplasia in premature infants: a meta-analysis with trial sequential analysis
Source: BMC Pulm Med. 2017 Dec 15;17:207. doi: 10.1186/s12890-017-0550-z (PMC5732371; doi:10.1186/s12890-017-0550-z)
Supplement: Supplementary file 2 — Outcomes measured in the 25 RCTs (DOCX 29 kb) [file 12890_2017_550_MOESM2_ESM.docx]

**Additional file 2: Table S2 Outcomes measured in the 25 RCTs**

| **Study** | | **Group** | | **Adverse outcomes** | | | | | | | | **Neurodevelopmental outcomes** | | |
| --- | --- | --- | --- | --- | --- | --- | --- | --- | --- | --- | --- | --- | --- | --- |
|  |  |  |  | **Sepsis** | **NEC** | **IVH** | **PVL** | **ROP** | **PDA** | **Hyperglycaemia** | | **NDI** | **CP** | |
|  |  | | **AACs vs placebo** | | | | | | | |  |  |  |  |
| Arnon^35^  1996 | | T  C | | 2  3 |  |  |  |  | 2  2 |  | |  |  | |
| Bassler^16^  2015 | | T  C | | 148  125 | 29  33 | 91  70 |  | 127  113 | 189  207 |  | |  |  | |
| Cao^36^  2016 | | T  C | |  |  |  |  |  | 3  4 | 4  3 | |  |  | |
| Cole^37^  1999 | | T  C | |  | 15  26 | 15  11 | 8  7 | 93  100 |  |  | |  |  | |
| Denjean^38^  1998 | | T  C | | 14  13 |  |  |  |  |  |  | |  |  | |
| Fok^39^  1999 | | T  C | | 6  7 | 7  8 | 3  3 | 3  1 | 6  4 | 17  20 | 12  14 | |  |  | |
| Giep^40^  1996 | | T  C | | 4  6 |  | 2  3 |  |  |  |  | |  |  | |
| Jangaard^41^  2002 | | T  C | | 15  12 |  | 3  1 | 3  4 | 2  3 |  |  | | 5  4 | 4  3 | |
| Jonsson^42^  2000 | | T  C | | 8  4 | 0  0 | 0  0 |  |  |  | 0  0 | |  |  | |
| Ke^43^  2016 | | T  C | |  |  |  |  |  |  |  | |  |  | |
| LaForce^44^  1993 | | T  C | |  | 3  3 |  |  |  |  |  | |  |  | |
| Merz^45^  1999 | | T  C | | 0  1 |  |  |  |  |  | 2  1 | |  |  | |
| Nakamura^24^  2016 | | T  C | | 7  14 | 2  5 | 17  18 | 1  5 | 42  41 |  |  | | 19  21 | 9  10 | |
| Pappagallo^46^ 1998 | | T  C | |  |  |  |  |  |  |  | |  |  | |
| Townsend^47^  1998 | | T  C | |  |  |  |  |  |  |  | |  |  | |
| Wen^48^  2016 | | T  C | |  | 4  3 |  |  |  | 18  19 | 5  3 | |  |  | |
| Yeh^49^ 2008  Kuo^50^ 2010 | | T  C | | 6  5 |  | 6  7 |  | 25  21 | 36  32 |  | | 11  13 |  | |
| Yeh^25^  2016 | | T  C | |  |  |  |  |  |  |  | | 26  34 |  | |
| Yong^20^  1999 | | T  C | | 10  5 |  |  |  |  |  |  | |  |  | |
| Zimmerman^51^ 2000 | | T  C | | 14  14 |  | 19  19 |  | 13  15 | 4  7 |  | |  |  | |
|  |  | | **Inhaled corticosteroids vs systemic corticosteroids** | | | | | | | | | | |  |
| Dimitriou^52^  1997 | | T  C | | 2  6 |  |  |  |  |  | 0  1 | |  |  | |
| Groneck^53^  1999 | | T  C | |  |  |  |  |  |  |  | |  |  | |
| Halliday^54^ 2001  Wilson^55^ 2006 | | T  C | | 93  92 | 25  24 |  |  | 19  15 | 152  132 | 33  64 | | 13  11 | 10  8 | |
| Rozycki^56^  2003 | | T  C | | 19  4 |  |  | 4  2 | 11  2 |  | 2  2 | |  |  | |
| Suchomski^57^ 2002 | | T  C | | 7  3 | 4  1 |  | 7  4 | 13  6 |  | 0  0 | |  |  | |

**RCTs: Randomized controlled trials, T: Treatment, C: Control, AACs: Airway administration of corticosteroids, IVH: Intraventricular hemorrhage, PVL: Periventricular leukomalacia, NEC: Necrotizing enterocolitis, ROP: Retinopathy of prematurity, PDA: Patent ductus arteriosus, NDI: Neurodevelopmental impairment, CP: Cerebral palsy.**
