# Supplementary figures and images for: Airway administration of corticosteroids for prevention of bronchopulmonary dysplasia in premature infants: a meta-analysis with trial sequential analysis
Source: BMC Pulm Med. 2017 Dec 15;17:207. doi: 10.1186/s12890-017-0550-z (PMC5732371; doi:10.1186/s12890-017-0550-z)

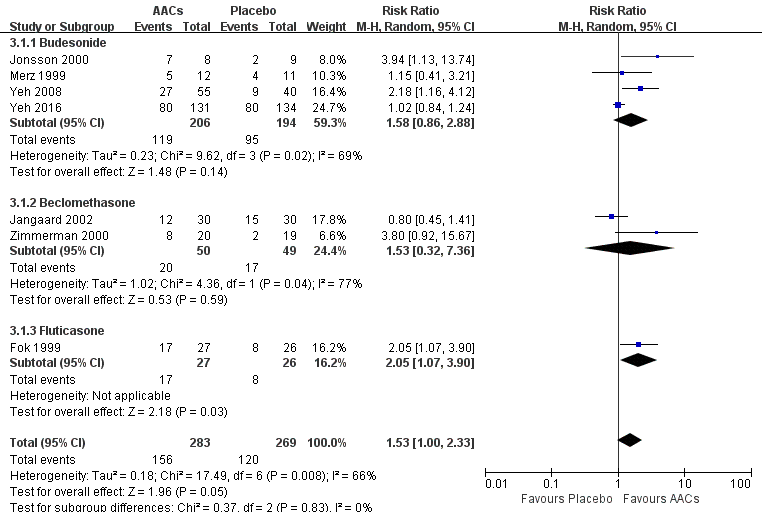

Supplement: Supplementary file 3 — Funnel plot of bronchopulmonary dysplasia, death, and requirement for systemic steroids with the use of AACs or placebo, AACs: Airway administration of corticosteroids. (JPEG 50 kb) [file 12890_2017_550_MOESM3_ESM.jpeg]

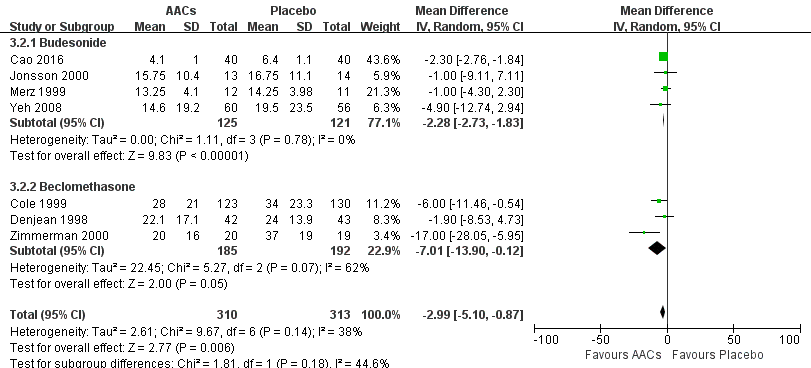

Supplement: Supplementary file 4 — Meta-analysis of requirement for systemic steroids with the use of AACs or placebo, AACs: Airway administration of corticosteroids. (JPEG 26 kb) [file 12890_2017_550_MOESM4_ESM.jpeg]

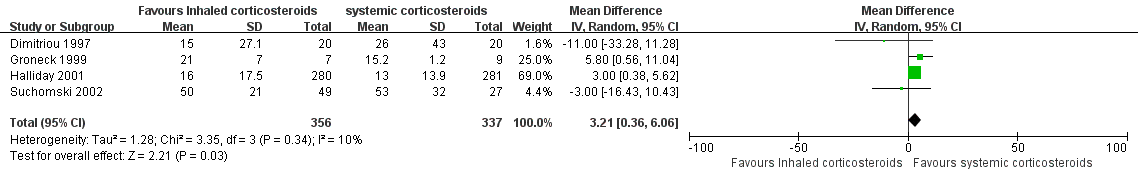

Supplement: Supplementary file 5 — Meta-analysis of success to extubate within 14 days with the use of AACs or placebo, AACs: Airway administration of corticosteroids. (JPEG 20 kb) [file 12890_2017_550_MOESM5_ESM.jpeg]

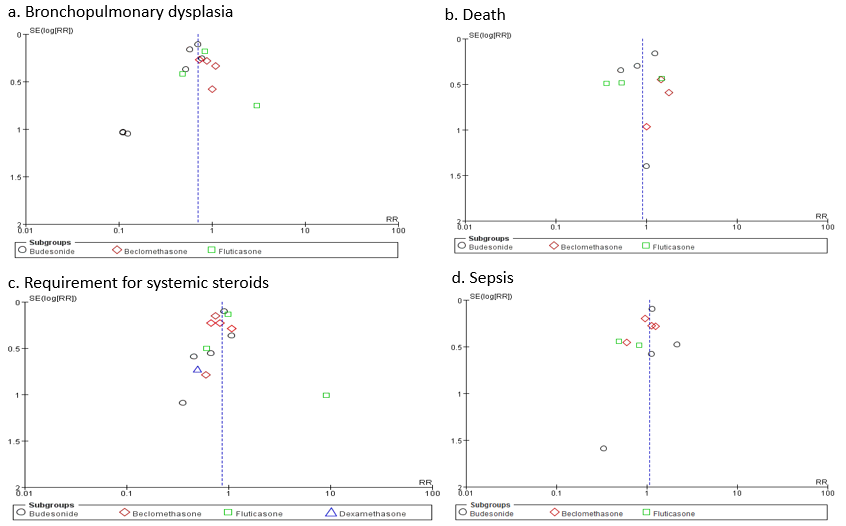

Supplement: Supplementary file 6 — Meta-analysis of duration of mechanical ventilation with the use of AACs or placebo, AACs: Airway administration of corticosteroids. (JPEG 17 kb) [file 12890_2017_550_MOESM6_ESM.jpeg]

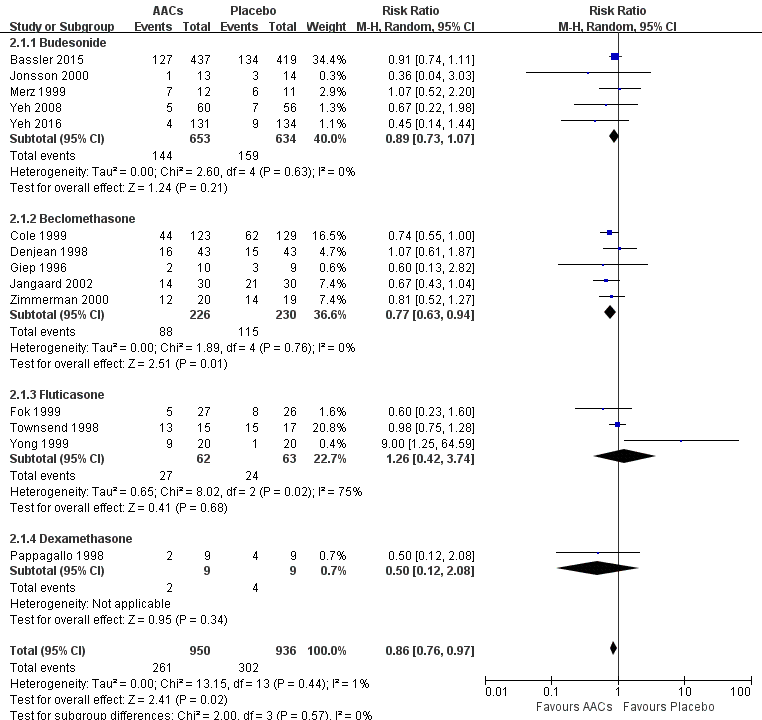

Supplement: Supplementary file 8 — Meta-analysis of duration of mechanical ventilation with the use of inhaled corticosteroids or systemic corticosteroids. (JPEG 11 kb) [file 12890_2017_550_MOESM8_ESM.jpeg]
