# Supplementary material for: Airway administration of corticosteroids for prevention of bronchopulmonary dysplasia in premature infants: a meta-analysis with trial sequential analysis
Source: BMC Pulm Med. 2017 Dec 15;17:207. doi: 10.1186/s12890-017-0550-z (PMC5732371; doi:10.1186/s12890-017-0550-z)
Supplement: Supplementary file 7 — Subgroup analysis of adverse and neurodevelopmental outcomes with the use of AACs or placebo (DOCX 17 kb) [file 12890_2017_550_MOESM7_ESM.docx]

**Additional file 7: Table S3 Subgroup analysis of adverse and neurodevelopmental outcomes with the use of AACs or placebo**

| Outcome or Subgroup | Studies | Participants | | Statistical Method | | | Effect Estimate |
| --- | --- | --- | --- | --- | --- | --- | --- |
| Sespis | 13 | 1547 | Risk Ratio (M-H, Random, 95% CI) | | | 1.09 [0.94, 1.26] | |
| Budesonide | 4 | 1023 | Risk Ratio (M-H, Random, 95% CI) | | | 1.16 [0.96, 1.40] | |
| Beclomethasone | 4 | 169 | Risk Ratio (M-H, Random, 95% CI) | | | 1.01 [0.78, 1.31] | |
| Fluticasone | 2 | 264 | Risk Ratio (M-H, Random, 95% CI) | | | 0.62 [0.33, 1.17] | |
| NEC | 5 | 1535 | Risk Ratio (M-H, Random, 95% CI) | | | 0.75 [0.54, 1.04] | |
| Budesonide | 2 | 1016 | Risk Ratio (M-H, Random, 95% CI) | | | 0.88 [0.56, 1.39] | |
| Beclomethasone | 1 | 255 | Risk Ratio (M-H, Random, 95% CI) | | | 0.60 [0.33, 1.08] | |
| Fluticasone | 2 | 264 | Risk Ratio (M-H, Random, 95% CI) | | | 0.71 [0.33, 1.52] | |
| Hyperglycaemia | 5 | 343 | Risk Ratio (M-H, Random, 95% CI) | | | 0.98 [0.61, 1.57] | |
| Budesonide | 4 | 290 | Risk Ratio (M-H, Random, 95% CI) | | | 1.55 [0.62, 3.86] | |
| Fluticasone | 1 | 53 | Risk Ratio (M-H, Random, 95% CI) | | | 0.83 [0.48, 1.43] | |
| PDA | 6 | 1304 | Risk Ratio (M-H, Random, 95% CI) | | | 0.89 [0.79, 1.00] | |
| Budesonide | 4 | 1212 | Risk Ratio (M-H, Random, 95% CI) | | | 0.91 [0.80, 1.03] | |
| Beclomethasone | 1 | 39 | Risk Ratio (M-H, Random, 95% CI) | | | 0.54 [0.19, 1.56] | |
| Fluticasone | 1 | 53 | Risk Ratio (M-H, Random, 95% CI) | | | 0.82 [0.57, 1.17] | |
| IVH | 9 | 1632 | Risk Ratio (M-H, Random, 95% CI) | | | 1.05 [0.85, 1.30] | |
| Budesonide | 3 | 999 | Risk Ratio (M-H, Random, 95% CI) | | | 1.21 [0.92, 1.59] | |
| Beclomethasone | 4 | 369 | Risk Ratio (M-H, Random, 95% CI) | | | 1.10 [0.57, 2.15] | |
| Fluticasone | 2 | 264 | Risk Ratio (M-H, Random, 95% CI) | | | 0.92 [0.53, 1.62] | |
| PVL | 4 | 575 | Risk Ratio (M-H, Random, 95% CI) | | | 0.92 [0.42, 2.05] | |
| Beclomethasone | 2 | 311 | Risk Ratio (M-H, Random, 95% CI) | | | 1.02 [0.46, 2.29] | |
| Fluticasone | 2 | 264 | Risk Ratio (M-H, Random, 95% CI) | | | 0.74 [0.05, 10.49] | |
| ROP | 7 | 1586 | Risk Ratio (M-H, Random, 95% CI) | | | 0.99 [0.89, 1.09] | |
| Budesonide | 2 | 972 | Risk Ratio (M-H, Random, 95% CI) | | | 1.08 [0.89, 1.32] | |
| Beclomethasone | 4 | 403 | Risk Ratio (M-H, Random, 95% CI) | | | 0.94 [0.83, 1.08] | |
| Fluticasone | 1 | 211 | Risk Ratio (M-H, Random, 95% CI) | | | 1.00 [0.71, 1.39] | |
| NDI | 4 | 474 | Risk Ratio (M-H, Random, 95% CI) | | | 0.83 [0.62, 1.10] | |
| Beclomethasone | 1 | 56 | Risk Ratio (M-H, Random, 95% CI) | | | 1.25 [0.37, 4.17] | |
| Budesonide | 2 | 239 | Risk Ratio (M-H, Random, 95% CI) | | | 0.78 [0.55, 1.11] | |
| Fluticasone | 1 | 179 | Risk Ratio (M-H, Random, 95% CI) | | | 0.87 [0.51, 1.51] | |
| Cerebral palsy | 2 | 246 | Odds Ratio (M-H, Random, 95% CI) | | 0.97 [0.43, 2.18] | | |
| Beclomethasone | 2 | 246 | Odds Ratio (M-H, Random, 95% CI) | | 0.97 [0.43, 2.18] | | |

**AACs: Airway administration of corticosteroids**
