# Supplementary material for: Airway administration of corticosteroids for prevention of bronchopulmonary dysplasia in premature infants: a meta-analysis with trial sequential analysis
Source: BMC Pulm Med. 2017 Dec 15;17:207. doi: 10.1186/s12890-017-0550-z (PMC5732371; doi:10.1186/s12890-017-0550-z)
Supplement: Supplementary file 9 — Subgroup analysis of adverse and neurodevelopmental outcomes with the use of inhaled corticosteroids or systemic corticosteroids. (DOCX 15 kb) [file 12890_2017_550_MOESM9_ESM.docx]

**Additional file 9: Table S4 Subgroup analysis of adverse and neurodevelopmental outcomes with the use of inhaled corticosteroids or systemic corticosteroids.**

| **Outcome or Subgroup** | **Studies** | **Participants** | **Statistical Method** | **Effect Estimate** |
| --- | --- | --- | --- | --- |

| Sepsis | 4 | 737 | Odds Ratio (M-H, Random, 95% CI) | 1.00 [0.63, 1.61] |
| --- | --- | --- | --- | --- |
| Necrotizing enterocolitis | 2 | 640 | Odds Ratio (M-H, Random, 95% CI) | 1.09 [0.62, 1.93] |
| Persistent ductusarteriosus | 1 | 564 | Odds Ratio (M-H, Random, 95% CI) | 1.29 [0.93, 1.80] |
| Periventricular leukomalacia | 2 | 137 | Odds Ratio (M-H, Random, 95% CI) | 0.82 [0.28, 2.40] |
| Retinopathy of prematurity | 3 | 564 | Odds Ratio (M-H, Random, 95% CI) | 1.37 [0.78, 2.39] |
| Hyperglycaemia | 4 | 732 | Odds Ratio (M-H, Random, 95% CI) | 0.44 [0.29, 0.69] |
| Cerebral palsy | 1 | 116 | Odds Ratio (M-H, Random, 95% CI) | 1.20 [0.44, 3.30] |
| Neurodevelopmental impairment | 1 | 126 | Odds Ratio (M-H, Random, 95% CI) | 1.14 [0.47, 2.77] |
